# Bioluminescent and fluorescent reporter-expressing recombinant Akabane virus (AKAV): an excellent tool to dissect viral replication

**DOI:** 10.3389/fmicb.2024.1458771

**Published:** 2024-11-14

**Authors:** Jingjing Liu, Fang Wang, Jiangang Zhao, Yinglin Qi, Jitao Chang, Chao Sun, Zhigang Jiang, Junwei Ge, Xin Yin

**Affiliations:** ^1^State Key Laboratory for Animal Disease Control and Prevention, Harbin Veterinary Research Institute, Chinese Academy of Agricultural Sciences, Harbin, China; ^2^College of Veterinary Medicine, Northeast Agricultural University, Harbin, China; ^3^College of Animal Science and Technology, Tarim University, Alar, China; ^4^Institute of Western Agriculture, The Chinese Academy of Agricultural Sciences, Changji, China

**Keywords:** Akabane virus, reverse genetics system, reporter-expressing virus, nanoluciferase, mWasabi, cell tropism

## Abstract

**Introduction:**

Akabane virus (AKAV) is a worldwide epidemic arbovirus belonging to the Bunyavirales order that predominantly infects livestock and causes severe congenital malformations. Reporter-expressing recombinant virus represents a powerful tool to characterize the viral biology *in vitro* and *in vivo*.

**Methods:**

In this study, we have successfully established a reverse genetics system for AKAV. The recued virus possessed similar growth characteristics to the parental virus *in vitro*. Moreover, the recombinant AKAV reporter viruses expressing nanoluciferase (Nluc) or mWasabi were constructed by inserting into S segment, named rAKAV-Nluc and rAKAV-mWasabi, respectively.

**Results:**

We investigated the virological characteristics of rAKAV-Nluc and rAKAV-mWasabi and found that rAKAV-Nluc displayed similar growth kinetics as the parental virus and could stably produce the nano-luciferase even after 10 rounds of serial passages. rAKAV-mWasabi also exhibited comparable growth kinetics and genetic stability as the parental virus. We further used the two reporter viruses to test the susceptibility of different cell lines to AKAV and found that cell lines derived from various host species, including human, swine, cattle, and monkey enables AKAV replication efficiently, accelerating our understanding of the AKAV cell tropism range.

**Discussion:**

Taken together, our established reverse genetics system for AKAV provides more convenient screening tools and can be used to study AKAV virulence and tropism, and to elucidate the molecular biology of AKAV.

## 1 Introduction

Akabane virus (AKAV) belongs to the *Orthobunyavirus* genus within the *Peribunyaviridae* family, serving as the etiological agent of arthrogryposis-hydranencephaly syndrome in cattle, sheep, and goats (Oya et al., 1961). AKAV exhibits teratogenic and lethal properties, resulting in substantial economic losses to the farming industry (Agerholm et al., 2015). Transmission of this virus primarily occurs through biting midges of the genus *Culicoides*, with its widespread distribution spanning Australia, Southeast Asia, East Asia, the Middle East, and Africa (Zeller and Bouloy, 2000). Furthermore, serological studies showed that AKAV antibodies could be detected in bamboo rat, horses, and pigs (Tang et al., 2017; Tzeng et al., 2022; Yang et al., 2008), suggesting that AKAV is widely distributed across various animal hosts globally.

AKAV is a single-stranded, negative-sense RNA virus. Its genome is composed of three distinctive segments: large (L), medium (M), and small (S) (Bishop, 1996). The M RNA segment encodes two envelope glycoproteins including Gn and Gc, as well as the non-structural protein (NSm). Gn and Gc form hetero-multimers that create spike-like projections up to 20 nm in length. These projections facilitate virus attachment to the surface of target cells (Ludwig et al., 1991). The non-structural protein M (NSm) plays a pivotal role in suppressing the host immune response (Barker et al., 2023). The L fragment encodes the viral RNA-dependent RNA polymerase (RdRp), while the S RNA encodes the viral nucleocapsid (N) proteins and non-structural proteins (NSs). RdRp is encapsulated into the virions, which plays roles in both viral transcription and genome replication. While, N proteins bind to the viral RNA segments, forming ribonucleoprotein structures. Each segment exhibits partially complementary 5′ and 3′ ends, potentially promoting the formation of a “panhandle” secondary structure, which could efficiently interact with RdRp (Barr et al., 2003).

The advent of viral reverse genetics has greatly advanced our ability to generate recombinant or modified viruses, offering invaluable insights into various facets of viral biology. Notably, recombinant viruses equipped with reporter proteins have emerged as powerful tools for probing the virus life cycle, discerning modes of viral spread *in vivo*, and screening antiviral agents. One pivotal milestone in developing the reverse genetical system for *Orthobunyavirus* genus occurred in 1996, when a reverse genetics system based on Bunyamwera virus was pioneered (Bridgen and Elliott, 1996). Given the frequent occurrence of AKAV in Japan, research on its reverse genetics has been actively pursued to aid in the development of vaccines and treatments. In 2007, the first reverse genetics system for AKAV based on the TS-C2 strain were established, subsequently, recombinant AKAV expressing enhanced green fluorescent protein (eGFP-AKAV) were developed as well (Ogawa et al., 2007; Takenaka-Uema et al., 2015, 2016). Since then, Takenaka-Uema et al.'s (2019) team has further optimized this reverse genetic system.

In this study, we developed a reverse genetic system using a clinical isolate AKAV/GX/2016, serving as a valuable tool to explore the fundamental mechanisms of AKAV pathogenesis. We further generated rAKAV strains expressing either fluorescent (mWasabi) or bioluminescent (Nluc) reporters. These reporter-expressing viruses showed similar replication kinetics compared to the parental virus. Consequently, we used these reporter viruses for the assessment of AKAV capability to infect various host cells and found that AKAV could infect the cell lines originated from different species. Coral-derived mWasabi is a relatively bright green monomeric fluorescent protein that performs well in protein chimeras, providing a bright and stable fluorescent signal that does not significantly interfere with the localization or function of the target protein. The protein is also tolerant to a wide range of protein fusions and subcellular microenvironments and is virtually harmless to live cells and easy to detect. In addition, Nluc has a high degree of physical stability and is much more tolerant to temperature, pH and urea. In cells, Nluc exists as a single molecular species with no post-translational modifications and is uniformly distributed without significant regional variations. The novel substrate furimazine produces higher light intensities than natural coelenterazine and is more stable with lower background autofluorescence. The combination of these properties allows Nluc to be widely used as a cellular reporter gene to produce highly sensitive signals. The stability of Nluc is not dependent on disulfide bonds, so the enzyme can be efficiently expressed intracellularly or extracellularly. In addition, the small size of Nluc makes it ideally suited for use as a protein fusion tag, allowing its own luminescence to be correlated with the dynamics of specific intracellular proteins. Nluc may also provide a unique opportunity for the development of protein complementation assays, where the small size, high luminescence, and structural stability are more advantageous. Overall, our optimized system enhances AKAV recovery efficiency, offering valuable tools for studying AKAV molecular virology and advancing next-generation AKAV vaccines and expression vectors.

## 2 Materials and methods

### 2.1 Cell, viruses, and antibodies

BSR-T7/5 cells (a BHK-21 derivative cell that stably expresses T7 RNA polymerase), African green monkey kidney cells (Vero E6), Madin-Darby ovine kidney cells (MDOK), Madin-Darby bovine kidney cells (MDBK), Madin-Darby Canine Kidney cells (MDCK) and human embryonic kidney 293T cells (HEK293T cells) were cultured in Dulbecco's modified Eagle's medium (DMEM, Gibco) supplemented with 10% fetal bovine serum (FBS, Gibco), 100 units/mL of penicillin, and 100 μg/mL of streptomycin at 37°C in a humidified 5% CO_2_ atmosphere. The Aedes albopictus mosquito cells (C6/36) was cultured in Minimum Essential Medium (MEM), supplemented with 10% FBS and 1% penicillin/streptomycin, at 28°C in a 5% CO_2_ atmosphere. The AKAV strain AKAV/GX/2016 was the parental virus for generating the reporter virus below. The anti-AKAV N mAb was generated in our laboratory. Furthermore, Alexa Fluor™ 568 goat anti-mouse IgG (H + L) was purchased from Invitrogen.

### 2.2 Construction of recombinant full-length cDNA clones

**Figure 1:**
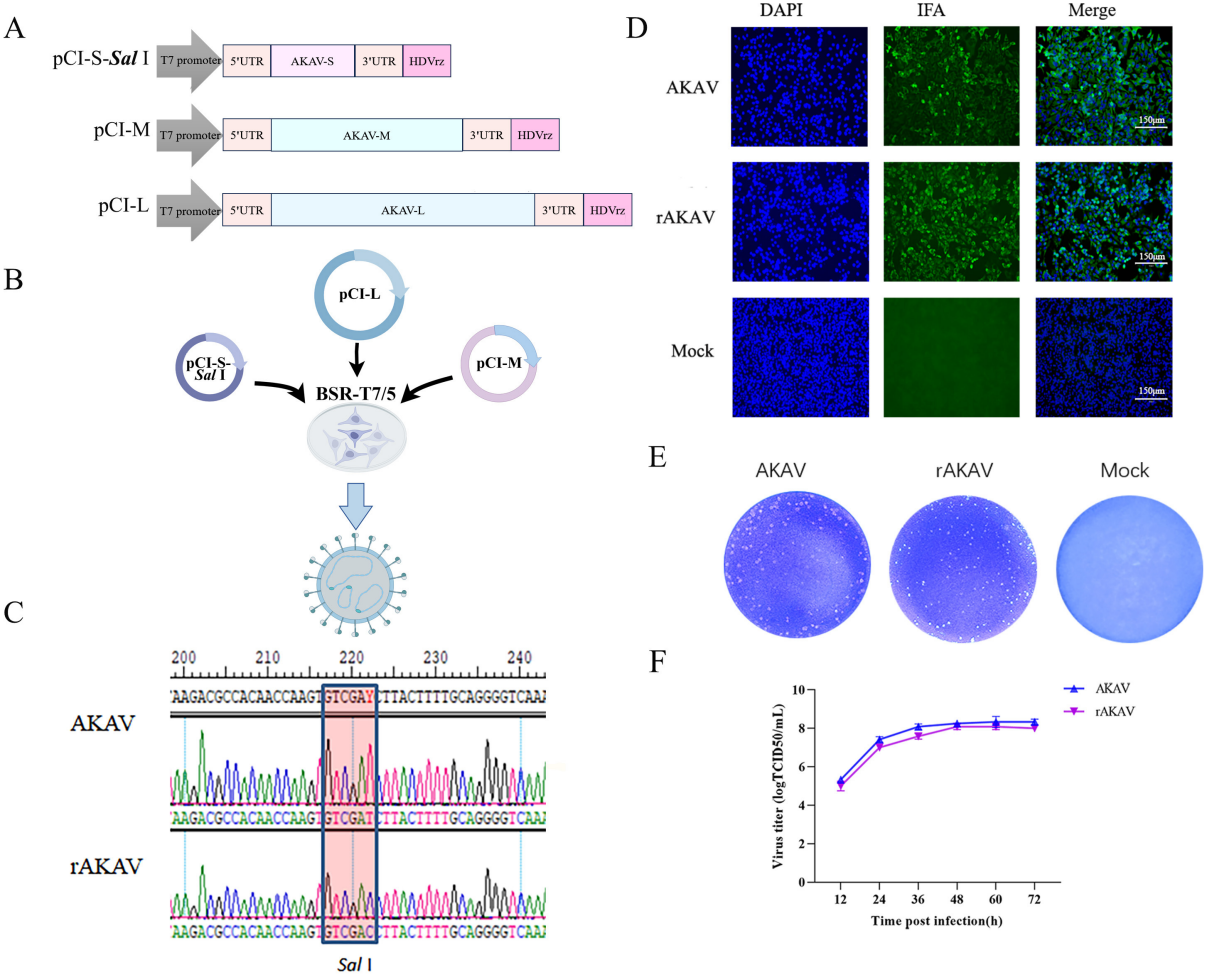
Construction, identification, and characterization of AKAV-WT and rAKAV. **(A)** The recombinant pCI plasmid of S segment, named pCI-S-*Sal* I. The recombinant plasmid of M segment, named pCI-M. The recombinant pCI plasmid of L segment named pCI-L. **(B)** Transfection strategy for rAKAV. **(C)** Sequence analysis of the amplified S products of AKAV. **(D)** Immunofluorescence assays of polyclonal antibody in the BSR-T7/5 cells at 24 hpi. **(E)** Plaque morphology of the two viruses in BSR-T7/5 cells at 72 hpi. **(F)** Growth kinetics curves of AKAV-WT, rAKAV in the BSR-T7/5 cells at MOI = 0.01. All the experiments were repeated three times.

**Figure 2:**
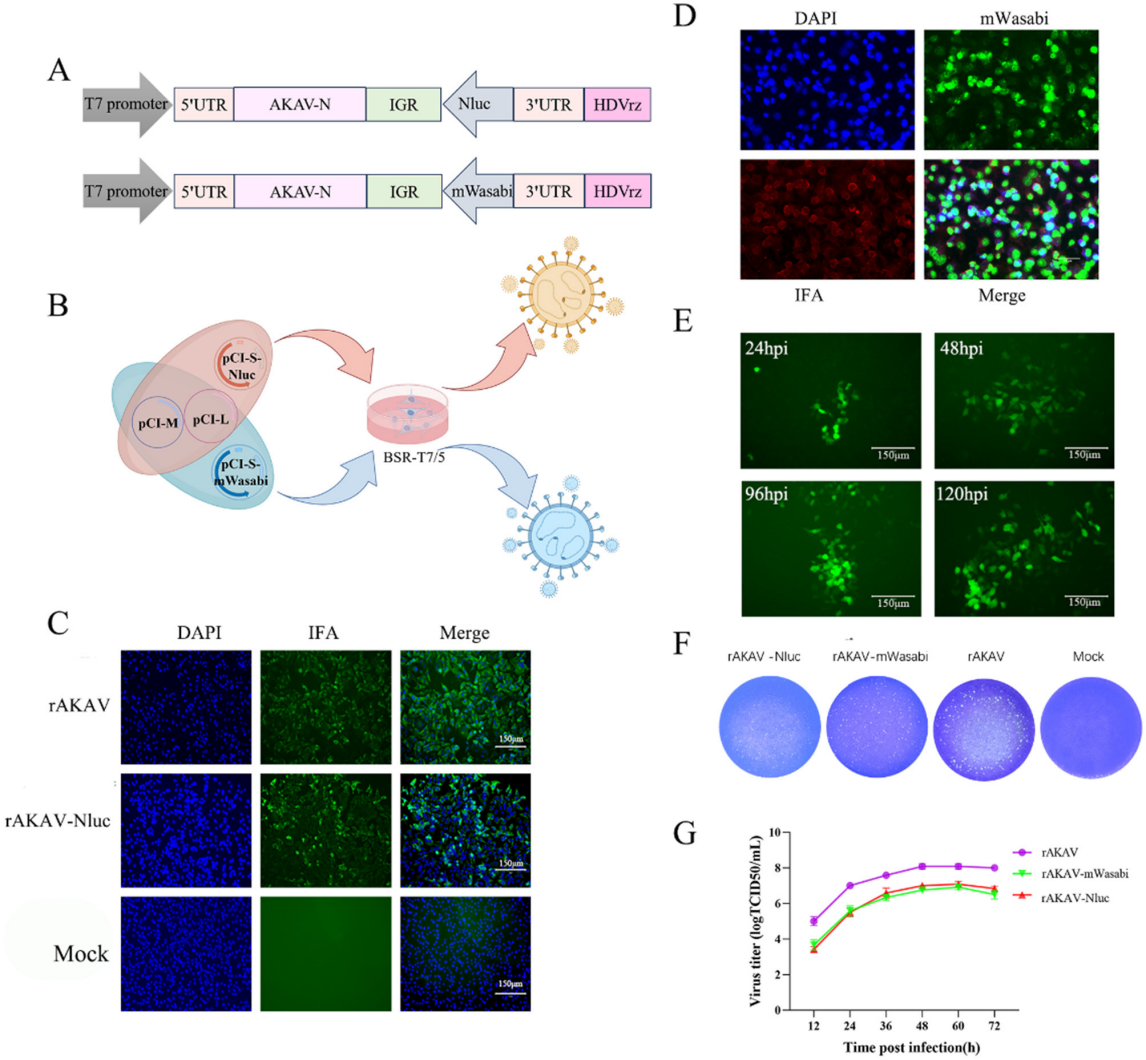
Construction, identification, and characterization of rAKAV-Nluc and rAKAV-mWasabi. **(A)** Schematic diagram of the intermediate plasmid pCI-S-Nluc and pCI-S-mWasabi. Based on pCI-S-*Sal* I, Nluc or mWasabi and IGR gene were added between 3′ UTR and N protein ORF, named pCI-S-Nluc and pCI-S-mWasabi. **(B)** Transfection strategy for rAKAV-Nluc and rAKAV-mWasabi. **(C)** Indirect immunofluorescence analysis of BSR-T7/5 cells infected with rAKAV and rAKAV-Nluc at 24 hpi using anti-AKAV N mAb. **(D)** Indirect immunofluorescence analysis of BSR-T7/5 cells infected with rAKAV-mWasabi at 24 hpi using anti-AKAV N mAb. **(E)** Changes in fluorescence at different times of rAKAV-mWasabi infection of BSR-T7/5 cells. **(F)** Plaque morphology and size of the rAKAV-Nluc and rAKAV-mWasabi cells are similar to that of the rAKAV. **(G)** Multiple-step growth curves of the rAKAV, rAKAV-Nluc and rAKAV-mWasabi on BSR-T7/5 cells. Cells were infected with rAKAV, rAKAV-Nluc and rAKAV-mWasabi at an MOI of 0.01, and cell supernatants were collected at the various time points post-infection, followed by TCID_50_ assays on BSR-T7/5 cells.

To construct a full-length cDNA clone from the S, M and L segments of AKAV/GX/2016, a set of primers was designed for amplifying the viral RNA genome using RT-PCR (Table 1). The complete cDNAs were amplified by PCR and cloned into the pCI-T7-HDVrz plasmid, which contains a T7 RNA polymerase promoter and HDV Ribozyme. Subsequently, the products were sequenced using Sanger sequencing. Three plasmids expressing viral RNAs were generated, namely pCI-S-*Sal* I, pCI-M, and pCI-L (Figure 1A). The pCI-S-*Sal* I plasmid was employed to generate pCI-S-Nluc and pCI-S-mWasabi, where the Nluc or mWasabi gene was fused with a Rift Valley fever virus-derived intergenic region (IGR) and inserted in the opposite orientation to the N gene (Figure 2A). The coding region for Nluc was amplified from pNL1.1 (NovoPro), while the coding region for mWasabi was amplified from pT7-mWasabi (Solarbio). This process resulted in the generation of an ambisense S-segment, designated S-Nluc and S-mWasabi, containing the N/NSs gene, IGR sequence, and either the Nluc or mWasabi gene.

**Table 1:** Nucleotide sequence of primers used in this study.

**RNA segment**	**Primer**	**Sequence (5^′^-3^′^)**
S	SF	TAATACGACTCACTATAAGTAGTGAACTCCACT
	SL	GATGCCATGCCGACCCTAAGTAGTGTGCTCC
M	MF	TAATACGACTCACTATAAGTAGTGAACTACCAC
	ML	GATGCCATGCCGACCCAGTAGTGTTCTACCAC
L	LF	TAATACGACTCACTATAAGTAGTGTACCCCT
	LL	GATGCCATGCCGACCCAGTAGTGTGCCCCT
L	L3590U	ATTCCGGGTAGTAACTTGTGC
	L3725L	CAGGGGAAGAAACTATCC
	L2085L	CAGATTAGCCATAATC
mWasabi	mWasabi-F	GGGCAGCCTTAACCTTTACTTGTACAGCTC
	mWasabi-L	CGGATGCCCAGGTCGGACCGCGAGGAGGTGGAGA TGCCATGCCGACCCTAAGTAGTGTGCTCCACTAATT AACTATAAACAATAAAATCCGAGCAGCTGAACAAAG TGTGCACCACATAGACATGGTGCACTTAGAAATAGA AGTAAGAAAACTGGAGAATCAGCAGAGAATGGTGA GCAAGGGCGAGG
Nluc	Nluc-F	GGGCAGCCTTAACCTCGCCAGAATGCGTTC
	Nluc-L	CGAGTGTGAAGACCATTCTCTGCTGATTCTCCAGTTT TCTTACTTCTATTTCTAAGTGCACCATGTCTATGTGGT GCACACCTTTGTTCAGCTGCTCGGATTTTATTGTTTAT AGTTAATTAGTGGAGCACACTACTTAGGGTCGGCAT GGCATCTCCACCTCCTCGCGGTCCGACCTGGGCAT CCGAAG
IGR	IGR-F	CTCAGTTGCCCAGATATCCTGGGCCAAATCTGGCTT CTCACCTGCAGCTAGAGCTTTCTTGGCTCAATTTGG TATTCAGATCTAAGTGGCTGCCCAGGGG
	IGR-L	AGGTTAAGGCTGCCCCAC

### 2.3 *In vitro* transfection

Recovery of Recombinant AKAV from Cloned cDNAs. Monolayers of BSR-T7/5 cells (8 × 10^5^) in 6-well plates were co-transfected with plasmids using 1.5 μL of Lipofectamine™ 3000 transfection reagent per microgram of plasmid DNA, as follows: 2 μg of each strain AKAV/GX/2016 rescue plasmids pCI-S-*Sal* I, pCI-M, and pCI-L (Figure 1B). After 120 h of incubation in FBS-free medium, transfected cells were lysed by freeze/thaw. The lysates were then transferred to fresh BSR-T7/5 cells. After adsorption at 37°C for 5 h, the lysate-adsorbed BSR-T7/5 cells were washed and cultured in FBS-free DMEM supplemented and incubated at 37°C for 48 h. When evident cytopathic effects (CPE) emerged, supernatants containing the recombinant viruses were harvested and named rAKAV.

For the rescue of the reporter virus, either pCI-S-Nluc or pCI-S-mWasabi, replacing pCI-S-*Sal* I, was included in the transfection process (Figure 2B). When CPE emerged and Nanoluciferase signal can be detected, supernatants containing the recombinant viruses were harvested and named rAKAV-Nluc. By the following day post-transfection, green fluorescence could be observed under a fluorescent microscope. The supernatants from the cell culture were collected at 120 h post-transfection, designated as rAKAV-mWasabi, and stored at −80°C.

### 2.4 Reverse transcription-PCR and sequencing

Viral RNAs were extracted from the supernatant of rAKAV-infected BSR-T7/5 cells using the SimplyP Total RNA Extraction Kit (BioFlux). Subsequently, the viral genome RNA was converted to cDNA using SuperScript II reverse transcriptase (TaKaRa). The S segments of the recombinant viruses were then amplified by PCR employing KOD FX Neo. Following amplification, the RT-PCR products underwent direct sequencing. The Nluc and mWasabi fragments were obtained using RT-PCR from the rescued reporter viruses and sequenced accordingly.

### 2.5 Indirect immunofluorescence assay (IFA)

The AKAV was diluted 50-fold with DMEM medium and 100 μL of the diluted virus was inoculated onto a 96-well plate covered with a single layer of BSR-T7/5, incubated for 1 h, then the inoculum was discarded and replaced with serum-free DMEM for further culture. 36 h post infection, AKAV-infected BSR-T7/5 cells were harvested, fixed and permeabilized using a mixture of methyl alcohol and acetone (1:1) and then washed with PBS once. The cells were blocked with 10% goat serum made in 1% BSA in PBS for 1 h, 100 μL/well, and then incubated with primary antibody (Mouse-derived positive sera; 1:500 dilutions made in 1% BSA in PBS), 100 μL/well. After 1 h inoculation at 37°C. Following three washes with PBS (5 min each, 100 μL/well), cells were incubated with secondary antibody (Alexa Fluor™ 488 goat anti-mouse IgG, diluted 1:2,000 in 1% BSA/PBS) for 1 h at 37°C (100 μL/well). Afterwards, the cells were stained with 4′6-diamidino-2-phenylindole (DAPI) for 15 min. After a final round of three washes with PBS (5 min each, 100 μL/well), results were observed using an inverted fluorescence microscope.

### 2.6 Growth kinetics and plaque morphology

A monolayer of BSR-T7/5 cells was infected with recombinant virus at an MOI of 0.1 PFU/cell for multi-step growth curves. After adsorption for 1 h at 37°C, cells were washed twice with PBS, and the medium was replaced with DMEM containing 0.9% agar and 5% FCS. Cells were frozen at 80°C at 12, 24, 36, 48, 60, and 72 h post-infection. Virus titers were determined in a TCID_50_ using BSR-T7/5 cells. Kinetics were evaluated based on data from three independently replicated experiments.

For the plaque assay, the BSR-T7/5 cells were infected with recombinant virus at a MOI of 0.001. The virus was allowed to adsorb to BSR-T7/5 cells for 1 h at 37°C, followed by two washes. Subsequently, the cells were covered with DMEM containing 0.9% agar and 5% FCS. After 3 days of incubation, the overlay was stained with 0.75% crystal violet, 10% formaldehyde and 5% ethanol.

### 2.7 Nanoluciferase and mWasabi stability during passaging

The rescued rAKAV-Nluc and rAKAV-mWasabi were serially passaged in BSR-T7/5 cells for 10 passages. The cell culture supernatants from passages F1 to F10 were harvested to extract viral RNA for RT-PCR analysis, assessing the stability of the foreign sequence in the rAKAV-Nluc and rAKAV-mWasabi genomes. The amplified products were analyzed using agarose gel electrophoresis and samples from passages F1, F5, F10, and F15 were subjected to Sanger sequencing.

### 2.8 Nanoluciferase activity assay and fluorescent protein observation

HEK293T, BSR-T7/5, Vero E6, MDOK, MDBK, MDCK and C6/36 cells were seeded in 96-well plates and infected with rAKAV-Nluc at a MOI of 0.01. After 48 h post-infection, the infected cells were lysed using an integrated lysis buffer and subjected to luciferase activity assay using the Nano-Glo Luciferase Assay System (Promega), following the manufacturer's instructions. Similarly, fluorescence in cells infected with the rAKAV-mWasabi virus was observed using inverted fluorescence microscopy after 48 h of incubation under similar conditions.

### 2.9 Statistical analysis

GraphPad Prism 9.5 software and Excel were employed for statistical analysis. The nano-luciferase activity data obtained from experiments were presented in graphics or a table as the mean and standard deviation (SD). Abbreviations for technical terms were provided upon their first usage. Each sample underwent luciferase activity measurement in triplicate, with the activity value at 0 h post-transfection or infection subtracted during calculations. For viral titration, whether through luciferase or endpoint dilution assays, we conducted three separate experiments, each in triplicate, for each recombinant virus, as well as the parental virus. All calculations were performed using Microsoft Excel.

## 3 Results

### 3.1 Establishment of reverse genetics systems for AKAV/GX/2016

To successfully rescue AKAV/GX/2016 using a T7 RNA polymerase-based reverse genetic system, we constructed a full-length cDNA clone from the S, M and L segments of AKAV/GX/2016. All these three plasmids expressing viral RNAs were confirmed by Sanger sequencing, namely pCI-S-*Sal* I, pCI-M and pCI-L. We then used two different transfection reagents, PEI (plasmid: PEI = 1:2, 1:3, 1:4) and Lipofectamine™ 3000 (plasmid: Lipofectamine™ 3000 = 1:1.5) for the recovery of AKAV in BSR-T7/5 cells that is permissive to AKAV infection. To further validate the efficiency of the rescue system, we also tried different transfection ratios and total amount of different plasmids [pCI-S-*Sal* I, pCI-M, pCI-L = 1:3:2.5 (total amount of plasmid = 0.65 μg) (Lowen et al., 2004), 1:1:1 (total amount of plasmid = 3 μg, 6 μg, 9 μg) (Chen et al., 2021), 2:1:2 (total plasmid amount = 3 μg) (Takenaka-Uema et al., 2016)]. Despite varying plasmid ratios and total amounts, we failed to rescue AKAV/GX/2016 by using PEI transfection reagent. Compared to other transfection reagents, Lipofectamine™ 3000 proved most efficient in transfecting larger plasmids into cells (Alencar et al., 2021). Surprisingly, we could successfully rescue AKAV/GX/2016 across all plasmid ratios and total amounts tested followed by Lipofectamine™ 3000 transfection. This suggests that while the plasmid proportions are not severely restrictive, transfection efficiency can be influenced by transfection reagents.

### 3.2 Characterization of rAKAV

By following the described protocol above, a significant CPE was observed in the transfected cells on day 3 post-transfection. The rAKAV was subjected to an immunofluorescence assay (IFA) to assess infectivity in BSR-T7/5 cells using anti-AKAV N mAbs. As expected, AKAV-specific green fluorescence was observed in cells infected with AKAV or rescued viruses (Figure 1D). Meanwhile the RT-PCR and DNA sequencing analyses of rAKAV both confirm the persistent presence of the *Sal* I molecular marker (Figure 1C). The plaques formed by rAKAV in BSR-T7/5 cells exhibited a similar size to the wild type (Figure 1E). Virus titer detection at 12, 24, 36, 48, 60, and 72 h, at the MOI = 0.01, revealed that the growth curves of rAKAV and AKAV followed similar trends, with nearly identical titers (Figure 1F). The viral titer showed the most rapid increase occurred between 12 and 24 h. It continued to rise steadily from 24 to 60 h, peaking at 60 h, and subsequently declined from 60 to 72 h. All these results demonstrated that the reverse genetical system of AKAV/GX/2016 was successfully established.

### 3.3 Characterization of rAKAV-Nluc and rAKAV-mWasabi

Recombinant AKAV engineered to express eGFP genes have been developed for monitoring viral distribution in mice (Takenaka-Uema et al., 2015). However, the eGFP signal produced from viral replication is too weak for detection *in vivo* imaging study (Takenaka-Uema et al., 2019). Therefore, in this study, we introduced mWasabi reporter that was 1.6-fold brighter than eGFP into AKAV genome (Kaishima et al., 2016). In addition, nano-luciferase gene was introduced to allow more efficient and convenient real-time visualization and quantification of AKAV replication. The pCI-S-*Sal* I plasmid in the three-plasmid system was replaced with a plasmid containing a reporter gene, leading to the successful rescue of reporter viruses rAKAV-Nluc and rAKAV-mWasabi (Figures 2A, B). Immunofluorescence assay (IFA) results revealed the rAKAV-Nluc and rAKAV-mWasabi could produce AKAV N proteins (Figures 2C, D), meanwhile rAKAV-mWasabi-positive cells could be directly identified under fluorescence microscope (Figure 2E). Plaques formed by rAKAV-Nluc and rAKAV-mWasabi in BSR-T7/5 cells closely resembled those of the wild-type virus, exhibiting uniform shapes similar to rAKAV (Figure 2F). To assess the replication kinetics of the rescued reporter viruses, multiple-step growth curves for rAKAV-Nluc and rAKAV-mWasabi were determined after infection of BSR-T7/5 cells at a low multiplicity of infection (MOI) of 0.01 PFU/cell. Growth curves of the rAKAV-Nluc and rAKAV-mWasabi had no significant difference, while their titers were lower than that of the parental rAKAV (Figure 2G). This indicated that a possible effect of the reporter gene on the replication characteristics of this strain. Overall, we generated rAKAV-Nluc and rAKAV-mWasabi which can efficiently express the reporter gene.

### 3.4 Stability of reporter genes from recombinant reporter viruses during delivery in BSR-T7/5 cells

**Figure 3:**
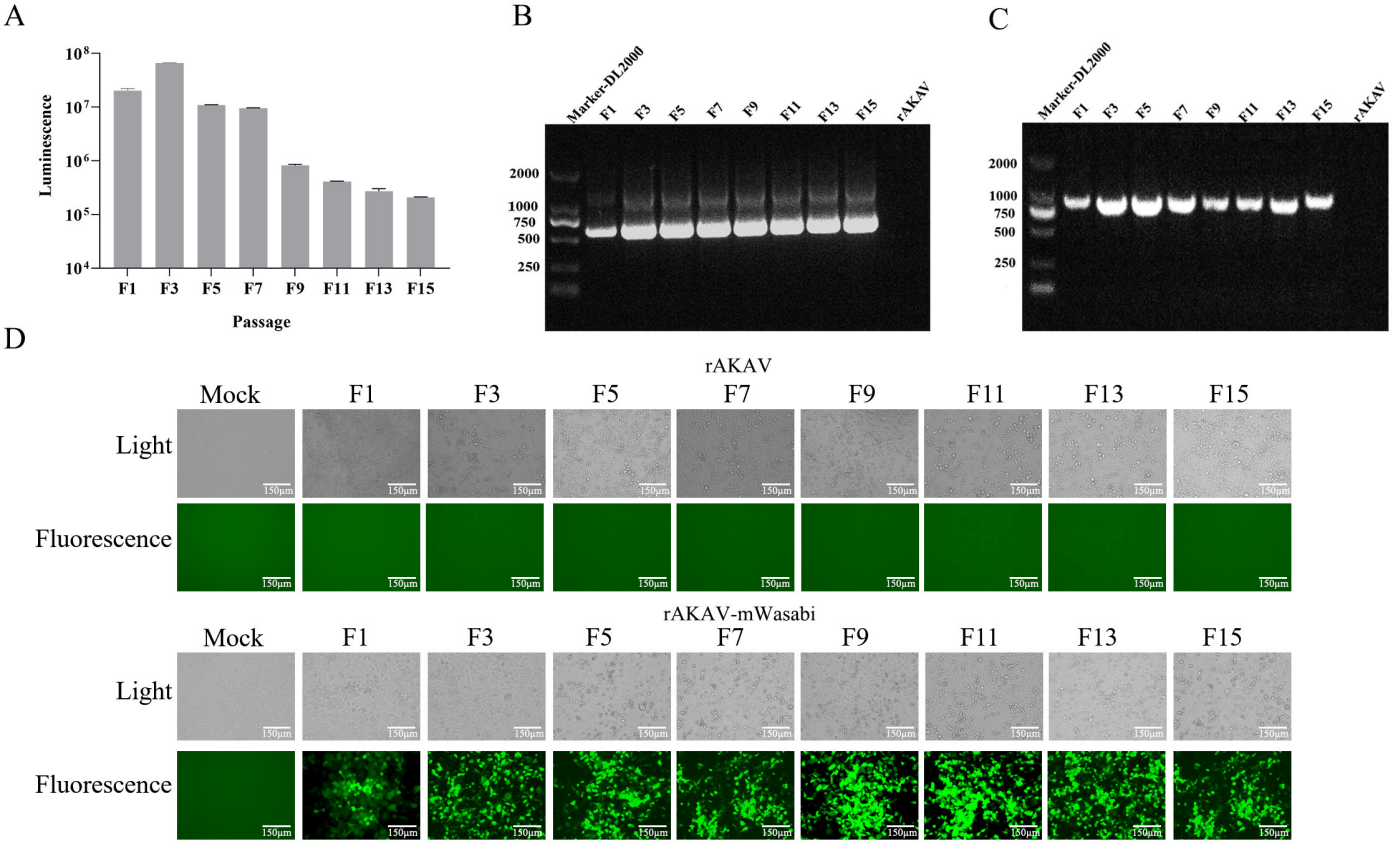
The reporter genes stability in rAKAV-mWasabi and rAKAV-Nluc during passaging in BSR-T7/5 cells. **(A)** The Nluc expression in BSR-T7/5 cells after infection with rAKAV-Nluc of F1-F10 generation. The expression of mWasabi was detected under a fluorescent signal at 48 hpi. **(B)** The RT-PCR detection of the Nluc gene in BSR-T7/5 cells after infection with rAKAV-Nluc of F1–F10. The resulting RT-PCR products were resolved by 1% agarose gel electrophoresis. **(C)** The RT-PCR detection of the mWasabi gene in BSR-T7/5 cells after infection with rAKAV-mWasabi of F1–F10. **(D)** The mWasabi expression in BSR-T7/5 cells after infection with rAKAV-mWasabi of F1, F3, F5, F7, F9, F11, F13, and F15 generation. The expression of mWasabi was detected under a fluorescent microscope at 48 hpi.

To evaluate the stability of rAKAV-Nluc in BSR-T7/5 cells, the recombinant virus underwent 10 serial passages. From F1 to F15, the luciferase signal intensity of rAKAV-Nluc virus was maintained above 10^5^ (Figure 3A). The culture supernatant of passages 1–15 virus was harvested for detecting the rAKAV-Nluc fusion gene by RT-PCR to determine the stability of the Nluc gene at the insertion site. The RT-PCR products with an expected 513 bp were detected from the supernatant of the infected BSR-T7/5 cells (Figure 3B). After nucleotide sequencing of the exogenous luciferase gene inserted region, the sequence alignment showed no mutant sites in the region of interest. The results confirmed the existence of intact Nluc in rAKAV-Nluc. Similarly, the stability of the reporter virus rAKAV-mWasabi was assessed through ten consecutive passages in BSR-T7/5 cells. Strong signals were observed from F1, F3, F5, F7, F9, F11, F13, and F15, indicating stable expression of the mWasabi gene during passaging (Figure 3D). Furthermore, Green fluorescence and cytopathic effects co-existed in BSR-T7/5 cells infected with rAKAV-mWasabi. RNA extracted from the infected cells at each passage stage and subjected to RT-PCR revealed a 711 bp band was consistently detected in rAKAV-mWasabi (Figure 3C). After nucleotide sequencing of the exogenous fluorescent protein gene inserted region, the sequence alignment showed no mutant sites in the region of interest. The data suggest that the reporter viruses remain stable and transmissible in BSR-T7/5 cells, with the fluorescence signal of rAKAV-Nluc virus was mildly maintained at a high level from F1 to F15. And the rAKAV-mWasabi maintained strong green fluorescence for 15 generations.

### 3.5 Application of Nluc/mWasabi reporter virus in identifying permissive cell lines

**Figure 4:**
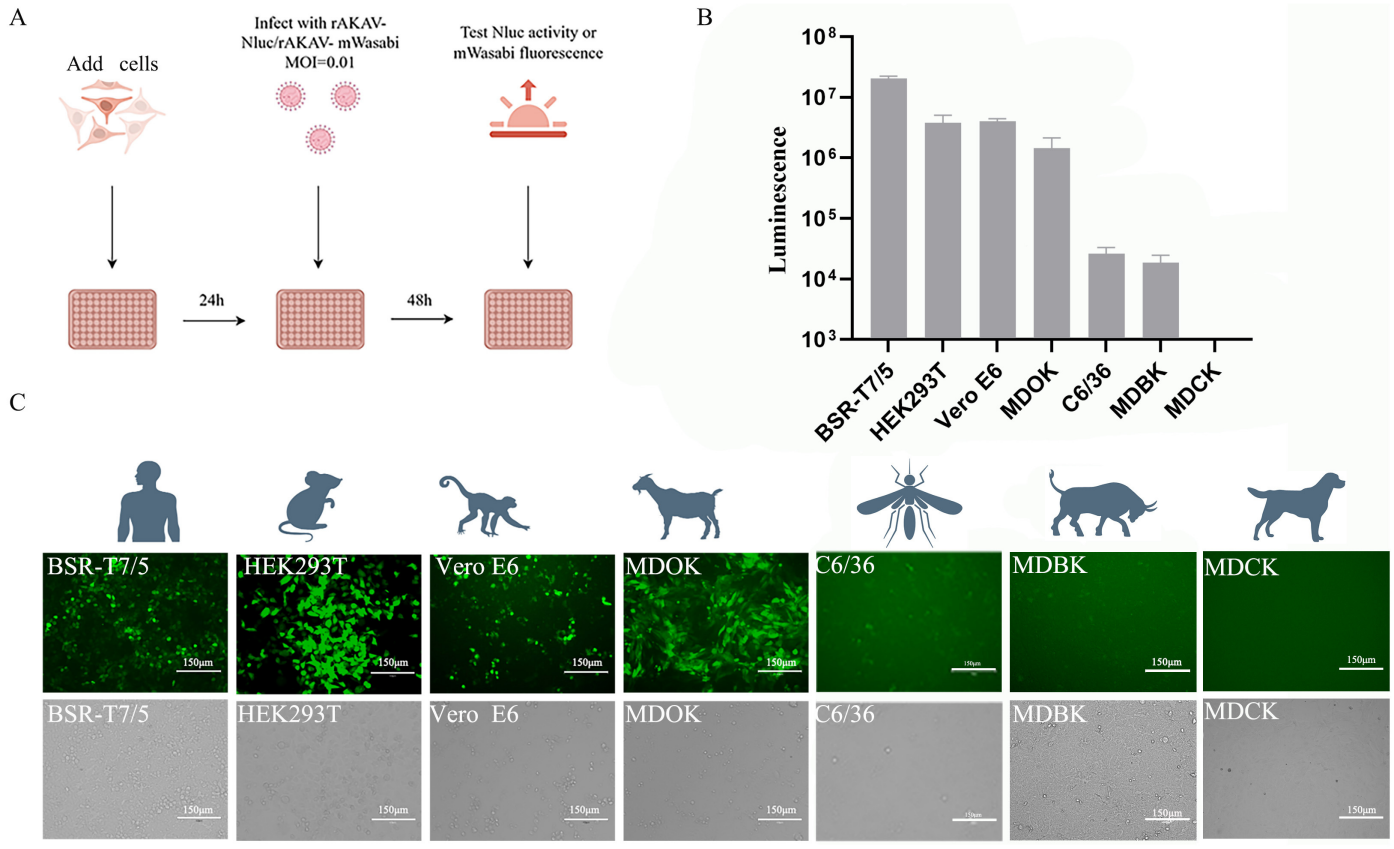
The infection of rAKAV-Nluc and rAKAV-mWasabi in cell lines derived from human, mouse, monkey, sheep, bovine and mosquito. **(A)** Strategies for reporting virus infection of different cell lines. **(B)** The Nluc signal in different cell lines infected rAKAV-Nluc at an MOI = 0.01 at 48 hpi. **(C)** The mWasabi expression in different cell lines infected rAKAV-mWasabi at an MOI = 0.01 at 48 hpi. All the experiments were repeated three times.

To explore the cellular tropism of AKAV, various cell lines derived from different animal species and humans were infected with rAKAV-Nluc and rAKAV-mWasabi at MOI of 0.01 (Figure 4A). Notably, MDOK, Vero E6 and BSR-T7/5 cells showed high susceptibility to the rAKAV-Nluc and rAKAV-mWasabi (Figures 4B, C), suggesting these cells were suitable for AKAV propagation, but lower infectivity in MDBK (bovine) and C6/36 (mosquito) cells. MDCK cells, which are not susceptible to AKAV, were used as negative controls, and we verified that the reported virus did not infect MDCK cells. Surprisingly, AKAV could efficiently replicate in HEK293T cells. Furthermore, MDBK cells displayed lower susceptibility to AKAV compared to HEK293T and Vero E6 cells, consistent with the infection pattern of the closely related SBV virus in MDBK cells (Elliott et al., 2013). In summary, the AKAV reporter viruses could be used for assessment of the replication pattern of AKAV in different cell lines.

## 4 Discussion

The Bunyavirales order is the largest group of RNA viruses. Among the 12 families within this order, Arenaviridae, Hantaviridae, Nairoviridae, Peribunyaviridae and Phenuiviridae are known to contain important pathogens that cause severe diseases in both humans and animals (Endalew et al., 2019; Oya et al., 1961). Understanding the life cycle of highly pathogenic viruses is often a prerequisite for developing effective vaccines and antiviral treatments. Therefore, there is an urgent need to develop robust tools to elucidate the molecular basis of pathogenesis. Reverse genetics technology has been extensively developed for bunyaviruses and is widely employed by researchers to dissect various aspects of the viral life cycle. As of now, reverse genetics systems for many members of the families Arenaviridae, Hantaviridae, Nairoviridae, Peribunyaviridae, and Phenuiviridae have been successfully established and applied to study a range of bunyaviruses (Caì et al., 2018; Ren et al., 2021, 2020; Tercero and Makino, 2020).

AKAV has shown its ability to cause stillbirths, abortions, and premature births in pregnant ruminants. The rising incidence of AKAV infection in ruminants is expected to lead to unpredictable and significant outbreaks (Oem et al., 2012). AKAV has been detected across various ruminant species worldwide. Moreover, epidemiological investigations conducted between 2005 and 2016 have found the virus was spreading in China (Wang et al., 2017). While some progress has been made in AKAV field, significant gaps remain, particularly in areas such as available vaccines and the host's antiviral innate immunity. To address this, we developed two reporter virus platforms: rAKAV-Nluc and rAKAV-mWasabi. These two-reporter gene-containing viruses serve as valuable tools for detecting and quantifying viral replication.

The utilization of AKAV reverse genetic systems remains somewhat restricted in constructing recombinant viruses for expressing foreign genes. Initially, fluorescence imaging was employed to localize the eGFP-based recombinant AKAV reporter virus in mice. To further optimize the detection method for eGFP, we explored the application of the mWasabi fluorescent protein in this study. Compared to eGFP, mWasabi fluorescent protein demonstrates superior fluorescence properties, boasting a relative brightness 167% greater than that of eGFP (Kaishima et al., 2016). Moreover, mWasabi exhibits higher sensitivity to acidic environments compared to eGFP, owing to its pK value (6.5:5.9). This implies that the green fluorescence of mWasabi is more susceptible to quenching in acidic lysosomes than that of eGFP (Chudakov et al., 2010). As a result, the fluorescence signal emitted by mWasabi proves to be more sensitive and accurate in assessing the localization of AKAV. Additionally, its low sequence similarity to eGFP may mitigate the potential impact of eGFP-mediated disulfide bond formation on virus rescue.

Additionally, we engineered a recombinant reporter virus (rAKAV-Nluc). Nano luciferase, being a novel luciferase, presents numerous advantages over Fluc and Rluc. Notably, it boasts enhanced stability, a reduced size, and a luminescence intensity surpassing 150 times that of the original luciferase (England et al., 2016). Secondly, rAKAV-Nluc offers time-saving benefits and increased sensitivity, making it suitable for neutralizing antibody assays (Yao et al., 2021). Furthermore, owing to the amplifying nature of the Nluc enzyme, rAKAV-Nluc exhibits a broader dynamic range and enhanced sensitivity compared to the rAKAV-eGFP viral assay. Additionally, reporter viruses have been extensively employed in screening interferon-stimulated genes (ISGs) and siRNAs to identify host factors that could impact viral replication (Karlas et al., 2010). Nluc has been employed in the study of viruses belonging to the Bunyaviridae family (Arenaviridae e.g.), including the thrombocytopenia syndrome virus (SFTSV) (Xu et al., 2024), among others (Wen et al., 2022). Leveraging *in vivo* imaging facilitates the visualization of virus distribution in diverse organs throughout the body, along with virus replication. Viruses harboring reporter genes integrated into their genomes serve as invaluable tools for detecting and quantifying viral replication, as well as for vaccine development.

One crucial application of the reverse genetic system is using the viral backbone for the expression of foreign genes. In a previous study with the Iriki strain, a recombinant virus containing the eGFP reporter gene was constructed, and it was inserted between the N and 3′UTR (Takenaka-Uema et al., 2016). Nonetheless, the reporter virus was exclusively employed to scrutinize AKAV tropism in mice. Previous studies on other bunyaviruses have suggested that the expression of foreign proteins, with varying insertion sizes, could potentially affect virus replication. In this study, we inserted reporter protein gene sequences between the N and 3′UTR regions in AKAV-S. Comparing the replication of rAKAV-mWasabi and rAKAV-Nluc with wild-type parental rAKAV, our findings illustrate that rAKAV-mWasabi and rAKAV-Nluc displayed comparable growth kinetics to the parental virus. In this study, we conducted cytophilic assays employing rAKAV-mWasabi and rAKAV-Nluc to evaluate AKAV capacity to infect various cells. AKAV enters non-bovine-derived cell lines (Vero and BHK cells) in the manner indicated Grid protein endocytosis. In contrast, AKAV infection in bovine-derived cell lines (MDBK cells) depends on dynamin (Bangphoomi et al., 2014). It may be that it is the two different mechanisms that contribute to the difference between AKAV-infected bovine and MDBK. The findings from these studies indicate that the reporter viruses exhibited diverse tropism properties across various cell lines of diverse origins. This offers valuable insights into the viral interaction with diverse host cells and may contribute to elucidating its pathogenesis. The discovery that AKAV infected HEK293T cells underscores the potential zoonotic risk linked to AKAV and underscores the importance of implementing suitable safety measures. Additionally, the establishment of AKAV reverse genetics systems has facilitated the generation of recombinant infectious viruses, which serve as powerful tools for understanding AKAV biology and vaccine development. While Akabane disease is widely prevalent in China, no vaccines are currently available. Therefore, AKAV reverse genetics systems provide a platform for the development of live-attenuated vaccine candidates.

## 5 Conclusion

Our study has successfully devised a reverse genetics system for AKAV and rescued two reporter viruses, serving as valuable tools for further AKAV research. These reporter viruses have demonstrated stability in cell culture and exhibited distinct tropism properties. While rAKAV-mWasabi facilitates qualitative virus analysis, rAKAV-Nluc is optimized for quantitative analysis. Together, these platforms complement each other. The reverse genetics platform we've established lays the groundwork for investigating the structure and function of AKAV proteins and can bolster the development of a live attenuated vaccine against AKAV. Leveraging reporter viruses enables the visualization and tracking of virus movement in infected animals, thereby enriching our comprehension of AKAV structure, function, replication, and pathogenesis. Consequently, this holds the potential to propel vaccine development and preventive strategies against AKAV infection forward.

## Data Availability

The raw data supporting the conclusions of this article will be made available by the authors, without undue reservation.
